# Remarks on the History and Treatment of Delirium Tremens

**Published:** 1832-04

**Authors:** John Ware

**Affiliations:** Fellow of the Massachusetts Medical Society.


### DELIRIUM TREMENS.

*Remarks on the History and Treatment of Delirium Tremens.* By JOHN WARE, M.D., Fellow of the Massachusetts Medical Society.

(Continued from page 207.)

MORBID anatomy has thrown no light upon the nature of that affection of the brain and nervous system, which gives rise to the peculiar symptoms of delirium tremens. Indeed, its history would rather lead us to expect that these symptoms do not depend on any organic changes discoverable by dissection, but merely on a disturbance in their functions. Accordingly, the morbid appearances, which have been observed, are not such as can account for the peculiar character of the disease. They are such as are common to many affections in which the brain is implicated. In four cases, and in two of these the disease had been accompanied by convulsions, I have seen effusion into the ventricles and upon the surface of the brain. Similar appearances have been frequently observed by others. But I am not aware that any other morbid appearances are recorded; and these, it will be obvious, do not at all account for the phenomena, but may be rather regarded as consequences than as causes.

Where delirium tremens has been complicated with other diseases, the morbid appearances which those diseases usually present will, of course, be exhibited. Since they do not, however, differ from those which the same diseases present in ordinary cases, it is necessary particularly to advert to them. It is desirable, however, to remark, in confirmation of some statements which have been before made, that in cases beginning with severe acute disease, and ending in a delirium which has entirely absorbed and obscured the symptoms of the original affection, there have been found, after death, morbid changes which prove incontestably that the original affection has continued, with unabated vigor, up to the very last moment of life. This has been particularly noticed in inflammations of the lungs and of the stomach.

The treatment of patients with delirium tremens is by no means confined to that period which has been designated as the paroxysm: since, from the symptoms of many cases of disease in intemperate persons, we are led to anticipate attacks of this sort, it is as important to prevent the paroxysm, or prepare for it, when we perceive its approach, as it is to conduct the patient safely through it when it is actually present. In speaking of the treatment, therefore, we are constantly to keep in view these two distinct inquiries:

1. By what measures may we prevent an attack of delirium tremens, when it is threatened?

2. By what measures may we arrest or alleviate the paroxysm, or carry the patient in safety through it?

With regard to the first inquiry, little can be said here, which may not be more intelligibly introduced hereafter. It may only be remarked in general, that whatever tends to alleviate or remove the symptoms with which the patient is first attacked, or to sooth and quiet the mind and nervous system, will contribute to the prevention of an attack of the delirium. There are, in fact, no direct means to be made use of, no remedies to be administered with this particular view. If the paroxysm is to be prevented, it is to be prevented by the judicious use of such general measures as will be spoken of in treating of its management.

There has been much uniformity of opinion among physicians concerning the object to the accomplishment of which the treatment is to be directed during the paroxysm: this object is the procuring of sleep. The absence of sleep is one of the most remarkable symptoms of the disease. When it terminates favorably, it terminates in sleep. It is not without foundation, therefore, that the treatment has had for its primary indication to bring about this termination. The patient, it has been emphatically said, "must sleep or die." There is no doubt that this is true; but may it not have been too hastily concluded from this undeniable position, that sleep must be procured by the assistance of art, or the patient will die. It is possible that the common impression which has been produced on our mind concerning this, is erroneous in two points of view: 1, we have concluded that sleep is the cause of the salutary change which takes place in the disease; and 2, that sleep, in whatever way induced, will have the same effect, and that it is therefore to be induced by artificial means.

In order to determine, concerning any disease, what influence our remedies actually exert upon it, we must first ascertain what will be its course and termination, if suffered to go through its usual series of changes without the interference of art. This is a point in the history of diseases to which reference should always be had in deciding upon the principles, or calculating the efficacy of the treatment to be employed. This is particularly desirable in those diseases, which, like that now under consideration, have but recently become the subjects of medical observation and inquiry.

I have witnessed a considerable number of cases of delirium tremens, in which the patient, after the establishment of the paroxysm, has been left to contend with it, without the administration of any remedy whose tendency was to cut it short, or in any decided way to modify its symptoms. The active treatment has been confined to the period of indisposition preceding the paroxysm; and after its accession articles of a negative character alone were administered, with the exception sometimes of purgatives. The result has uniformly been, that the disease has gone through that regular course, which has been already described in the former part of this paper, and has terminated in the manner there described, at a period seldom less than sixty or more than seventy-two hours from the commencement of the paroxysm.

The termination in these cases has also been almost uniformly favorable, except where there has been a combination of the delirium with some acute disease in itself dangerous, or where it has appeared in connexion with some fatal chronic malady. This course has been pursued, I do not mean to say without any deviation, but without any deviation which I believe to have essentially affected the result, in about fifty cases of the several classes which have been described; and although several deaths have taken place among them, none are recorded, except among cases which I have arranged, whether justly or not, in the third and fourth classes.

It may be stated, in confirmation of the opinion now expressed concerning the natural tendency of the paroxysm to terminate in a spontaneous and salutary sleep at the end of a certain period, that, even in the reports of cases which have been submitted to the public as evidences of the efficacy of various modes of practice, sleep has not actually taken place sooner than it would have done in the natural course of the disease, if the history which has now been given of it be founded on correct observation. In the cases which I have formerly treated with opium, and which have at last terminated well, a salutary sleep has not actually taken place till toward the close of the third day, let the quantity of opium be what it would. I have, indeed, seen sleep induced by opium at an earlier period, but it was premature; it passed into a state of coma, and the patient died.

I am satisfied, therefore, that, in cases of delirium tremens, the patient, so far as the paroxysm alone is concerned, should be left to the resources of his own system; particularly that no attempt should be made to force sleep by any of the remedies which are usually supposed to have that tendency; more particularly that this should not be attempted by the use of opium. I do not undertake to say that it can be never right to administer opium for the removal of the paroxysm itself; but I believe it can be rarely necessary, and I have not yet seen a case in which I think that it was.

It is no doubt very difficult to compare the success of the practice of different individuals; it is even difficult for an individual to compare the success of different modes of practice in his own hands at different times. The first cases of delirium tremens which occurred to me were treated exclusively with opium in large doses, then (in 1817) the popular practice of the day. The proportion of fatal cases was such as to satisfy me, in no very long time, that, if this remedy did no harm, it certainly could do little good. Subsequently the emetic practice, introduced by Dr. Klapp, came somewhat into favor, and though by no means uniformly successful, and never, so far as I know, cutting short the disease after it was once fairly established, it was far more satisfactory in its results than the treatment by opium. Various other remedies have been proposed, which I have either employed, or have known the effects of in the practice of others; but with none has the success been any greater than with the expectant mode of practice. I do not mean to claim even for this a success so remarkable as that which has been claimed by some writers for the practice with opium. I would only state what has been the impression produced by a very considerable number of cases on my mind.

No doubt the most prevalent opinion of physicians, with regard to the proper mode of treating delirium tremens, is in favor of the administration of opium in such doses as will procure sleep; yet, as has been already suggested, other plans have been proposed, and are preferred by a very considerable number; whilst many highly respectable practitioners still profess themselves unsettled in opinion concerning its proper management. A diversity of opinion concerning the treatment of any disease, may be generally considered as indicating some uncertainty with regard to the efficacy of any of the measures employed. Physicians in actual practice have not been uniformly satisfied with the degree of success which has attended the administration of opium, or they would not have been frequently searching, as has unquestionably been the case, for other remedies. Notwithstanding the almost unlimited success which has been claimed for the opium practice by some who have written in its favor, physicians in general do not find it equally successful, or they would not seek for any thing better. A specific remedy, easily administered, attended with no unpleasant accompaniments, and generally successful, would never be thrown aside, even a moment, for the sake of trying experiments with new remedies. We never hear the efficacy of sulphur in the itch doubted: it is true new remedies are sought for, not because sulphur is an uncertain, but because it is a disagreeable, remedy. There could be no such reason, if opium were a specific, or a tolerably successful remedy, in delirium tremens. Its exhibition is easily managed; practitioners have not been disappointed in its efficacy because they have failed to administer it rightly, but because they have not found it to answer the expectations which the representations of its advocates excited. The uncertainty of opinion among physicians, therefore, concerning the use of opium, and indeed generally concerning the proper treatment of delirium tremens, is a practical proof that no one method has any superior or exclusive claim to our confidence; or, at any rate, that opium does not command the great success which has been attributed to it.

So far from being beneficial, I believe there is ground to believe that the effect of opium, given during the paroxysm, is to increase the violence of the delirium, to produce a tendency to convulsions, to prevent the termination by a natural and salutary sleep, or to throw the patient into a state of of coma from which he does not awake. I do not mean to say that these effects are often produced. There is in this disease, as in some others, a happy insensibility of the system to the action of remedies, which allows it, in a large majority of instances, to take its own course essentially unaffected by them. It may seem presumptuous to make this statement in the face of such authorities as have written on this disease: but many, if not all, have taken it for granted that it is to be treated by medicine; they have never trusted to the spontaneous efforts of nature for a cure. Having pursued a different course myself, I am prepared to say, that there is the difference pointed out above between the cases which I have treated without opium, and those which I have treated with it myself, or of the treatment of which by others I have seen accounts.

But I have also feared that the unfavorable effects of opium have not been confined to its administration during the paroxysm. It has happened, in several instances, that the symptoms of the paroxysm have manifested themselves in cases where they had not been particularly apprehended, very soon after the exhibition of opium. I do not mean to say, that I have evidence to shew that opium is in all cases injurious in the diseases of the intemperate, or that it should never be employed: there are sometimes symptoms which absolutely demand its use, even at the risk, if there be any, of inducing delirium tremens.

The cases where delirium tremens seems to have followed directly on the use of opium, have been those chiefly in which it has been employed to check or control some dangerous or inconvenient symptom. Thus it has come on in cholera, and in the vomiting of drunkards from irritable stomach, soon after opium has been given to subdue the violent symptoms; or in dysentery and diarrhœa, after it has been used to stop the evacuations from the bowels; and this, whether it has succeeded in effecting the intended object or not. This coincidence has happened in so many cases as to lead to a suspicion that it might be something more than accidental.

It will follow, from what has been said, that we derive no advantage from any direct attempt to produce sleep, and thus to cut short the paroxysm. In what, then, is our treatment to consist? Are we to leave the patient wholly to the resources of nature and his constitution, or are we to endeavour to promote indirectly that salutary termination which we have no means of bringing about directly? It is not intended to imply, that we can do nothing for the disease in any way, or at any period. Something may be done to carry the patient safely through it, and sometimes to prevent it.

When the attack of delirium tremens is preceded by acute disease, the treatment in the first stage will be governed by precisely the same laws as those which direct us in ordinary cases. That course which is most likely to relieve the original disease, is most likely to prevent the attack of delirium, or if it do not prevent it, to make it run through its course in safety. The great danger in these cases arises from the complication of the severe disease of an important organ, with the unbalanced and irritated state of the nervous system. The only precaution to be taken, when we apprehend this result, is to effect the cure of the original affection with as little reduction of strength as possible, (and this is a precaution necessary to be taken in all cases of disease in the intemperate,) and, when possible, to avoid the administration of opium for the alleviation of symptoms which are usually benefited by it.

But with regard to the delirium itself, we shall best convey a view of the principles which are to guide us in its treatment, by passing briefly in review the remedies which are applicable under different circumstances to patients who labour under, or who are threatened with it.

Bloodletting is a remedy which the symptoms would, on a first view, suggest as highly appropriate. It has accordingly been adopted, and at first indiscriminately, without reference to the nature of the case, or the stage of the disease. Hence its reputation has fallen much lower than it really deserves. It may be often employed with great advantage, and it probably succeeds more frequently in preventing or mitigating the paroxysm, than any other remedy, emetics alone perhaps excepted. It is very satisfactorily shewn by Dr. Hayward, in an examination of Dr. Sutton's work on Delirium Tremens,* that all the cases which were treated with opium, and whose successful termination was attributed to the use of that drug, had been bled at some stage of their progress, either before the occurrence of the delirium, or very soon after its access. This accords with such observations as I have been able to make. The most remarkable cases in which the symptoms of approaching delirium tremens have subsided without its occurrence, have been when bleeding, either generally or locally, has been adopted.

In resorting to general bloodletting, we should, of course, be much governed by a regard to the constitution, previous health, &c. of the patient. Old and worn-out drunkards would probably be always the worse for it; but there are few other cases in which it will be injurious in the early part of the disease. In cases where the pulse is hard, strong, and not very rapid, where there is some pain in the head, with a flushed countenance, and a skin rather dry than moist, venesection is particularly indicated, and will serve to mitigate or prevent the attack. The objections to this remedy seem most likely to have arisen from its employment in too advanced a period of the disease; it is then only that it can be absolutely dangerous. It would seem almost evident, before experience, that if presevered in with a view to the removal of the paroxysm, after it has been once fairly established, and after the skin has become moist and flabby, the pulse rapid and weak, and after all the symptoms indicate a state of high irritation,

* New England Journal of Medicine and Surgery, vol. xi. 1822.

it must be injurious, if not fatal. It is probably this use, or rather this abuse of the remedy, which has led writers to denounce it in unqualified terms.

Local bleeding is more universal in its adaptation to delirium tremens, and may be employed in a majority of cases. It appears to have an effect to render more safe, and sometimes to prevent the paroxysm. I know how liable we are to over-rate the effects of remedies, of whose efficacy we have formed a favorable opinion; and it may be so with regard to this. But I believe that there is decided benefit in most cases, in bleeding from the head and neck, by leeches and cupping, at any time before the paroxysm, or during the first day of it. I have even employed it on the third day with the apparent effect of hastening the favorable termination. This was in the case of a man of sixty years old, of a highly irritable temperament, in whom the disease was accompanied by no local affection. On the evening of the third day, when he was violently delirious, the application of twenty leeches was followed by an immediate mitigation of the symptoms, and in a very short time he fell asleep. It is not to be inferred that in this case the loss of blood had any influence on the final issue of the case. It would probably have been terminated in the same way in the course of a few hours by the spontaneous efforts of the system; but the decided and direct effect of the remedy was such as to shew the influence which it is capable of exercising.

In a large proportion of cases of delirium tremens, the digestive organs are deranged in the same way that they are usually found in intemperate patients, with whatever disease they may be affected. This is often the only disorder which can be detected previously to the attack of the delirium. To remedy this state of the digestive organs, emetics may be administered with great benefit.

They have also been recommended as a means of cutting short the disease and inducing sleep, after the paroxysm has been fairly established. It has been supposed that there is a morbid condition of the stomach in patients with this affection, which occasions the disorder of the brain and nervous system; and that powerful emetics remedy this condition, and prevent or cut short the paroxysm. It is not impossible that this is the case, and the authority in favor of the practice is highly respectable. Yet the evidence is not sufficient to shew that the accession of sleep is hastened by their administration. So far as I have tried emetics, though, as above stated, they may have had a favorable effect on the digestive organs, and improved the general state of the system, when given before the paroxysm, sleep has not been produced at a period anterior to that at which it were to have been otherwise expected.

No particular advantage arises from purging carried to any great extent. It is desirable in the beginning of every case, unless the bowels are in a perfectly natural state, to clear the alimentary canal thoroughly by some active cathartic; and afterward to maintain it in an open state by gentle laxatives. It is also necessary, where the secretions are in a depraved state, to correct them by the use of the common alteratives. After the accession of the paroxysm, no medicines of this kind have any appreciable effect in modifying its course.

The use of blisters has been reprobated as tending to increase the severity and danger of this disease. The dread of them seems to have been handed down to us from one of the first writers who has treated of it; for it does not appear that they have been since much employed. It cannot be affirmed that they are usually found of any decided advantage; but having very frequently employed them, I am prepared to assert that they do no harm, and, so far as their irritation is an objection to their use, that there are few diseases in which they produce so little. I recollect one example which well illustrates their innocence, at least, if not their efficacy. A man was brought into the Boston Almshouse, for an abscess around the knee-joint, preceded by severe inflammation, which was the consequence of external injury. Delirium tremens supervened. The patient had been bled, and the emetic, and afterwards the opiate, practice, were put in operation upon him to their full extent. The day preceding the night when the paroxysm had a favorable termination in sleep, three large blisters were applied, one on the back of the neck, and one on the upper part of each arm; all of which took effect. At the time I attributed the favorable result to the vesication, since it came on so directly afterward; but, according to subsequent observation, it did not take place sooner than is usually the case in patients who recover. It is, however, sufficient to shew that the very extensive employment of blisters does not prevent, even temporarily, the salutary termination in sleep, since the irritation of the remedy was here at its highest point at the very time when the patient became quiet and slept.

Mercurials have not, I suspect, been often given in delirium tremens, with any view to their specific effect on the system: the disease is too rapid in its progress to allow time for a ptyalism to take place. In a single instance it was produced by large doses of calomel and opium, in an individual who had been subjected to the operation of active emetics in the early part of his disease. A favorable sleep took place about the time that the mouth became sore; but no earlier than it may be expected in those cases which have not been treated by medicine.

The warm bath has been suggested as a remedy in this disease. I have never used it, and cannot therefore bear witness to its efficacy, but it is likely to do good during the period which precedes the paroxysm. During the paroxysm itself, there can be no benefit which would in any degree compensate for the confusion, trouble, and exposure which must be occasioned by it.

Various other remedies have been from time to time proposed, and their efficacy supported by the narration of cases in which sleep has been supposed to follow as a consequence of their use: among these remedies are assafœtida, digitalis, hyoscyamus, valerian, prussic acid, sulphuric ether, tincture and infusion of hops, infusion of wormwood, and borax. Of nearly all these remedies I have made trial in one or more cases, and some of them I still continue to exhibit: but I have found no reason for attributing any efficacy to their operation in hastening the termination of the paroxysm.

Having premised these observations upon the general applicability of these remedies to the treatment of delirium tremens, we may close this account of the disease by stating, in a summary manner, the course which should be pursued in its management, according to the principles which have been laid down.

Where we are satisfied that the delirium is the immediate consequence of the excessive use of liquor in an individual previously in good health, no medical treatment is necessary. If the patient be left to himself, and be debarred from ardent spirits, the attack subsides spontaneously: in the worst cases, no medicines can be required beyond a dose of salts, and an infusion of valerian, of wormwood, or of hops.

In those cases which are preceded by some general derangement of the system, without any well-defined disease, our course is to be determined by the nature of the derangement and the state of the constitution. Where the patient is robust and vigorous, more particularly where, in such a patient, there has been convulsions or severe pain in the head, general bleeding should be freely adopted, and is the most important remedy. In almost all cases, let the constitution be what it may, local bleeding may be regarded as beneficial, if not indispensable; and it is particularly called for where there is dizziness, pain in the head, or much flushing of the countenance, with heat in the head and face.

When the digestive organs have been long in a deranged state, especially when the stomach appears to be loaded with a mass of secretions which are offensive to it, and which excite it to ineffectual vomitings, a powerful emetic of tartarized antimony is of essential benefit. In common cases a combination of ipecacuhana with the sulphate of copper or of zinc, is sufficient for the proper evacuation of the stomach. This may be followed by a cathartic of calomel, either combined with, or followed by some other article which will promote its full operation. It is afterwards only necessary to regulate the bowels by mild laxatives, unless some unusual symptom arise, which indicates a more active evacuating treatment.

This course, with the exception of general bleeding, may be pursued whether the physician be called before or soon after the commencement of the paroxysm. Little else is required in the large majority of cases. Particular symptoms may call for the administration of particular remedies; but of such a necessity, a judgment must be formed in each case on a consideration of the general principles upon which the disease is to be treated.

Still, although there are no particular remedies which appear to have any influence in the removal of the disease, there are many articles which I have been in the habit of prescribing, which do not interfere at all with the regular course of the disease, and have a grateful and supporting operation upon the system. Such are the infusion and tincture of valerian and of hops: an effusion of chamomile; a solution of carbonate of soda, or of ammonia, or of sulphate of quinine, &c.

The use of spirituous liquors during delirium tremens, has been recommended as a means of promoting sleep. If I believed them really advantageous in this respect, I should hardly feel justified in resorting to them, since this must diminish what little prospect there may be, that the disease will be a means of breaking up the habit of intemperance. I have accordingly never recommended this practice, and if the views which have been presented of the course of the disease be founded on correct observation, it will appear probable that it has not had the influence which has been attributed to it.

The diet should, in most cases, consist entirely of nutritious liquids: but occasionally the appetite remains good, and the patient may be then indulged with small quantities of his ordinary food, particularly in the morning.

As little restraint should be exercised over the patient as is consistent with the circumstances of the case. It is usually necessary, from considerations of propriety, to confine him to his house, and perhaps to his room, but within the determined limits he should be as little controlled as possible. When circumstances admit, it is better that he should have free range within doors and without; the precaution being always taken of having some able-bodied person to watch his motions. There is little to fear from exposure; patients have been frequently exposed to the night air, to cold, and to rain, for hours, and with very insufficient clothing, without the slightest injury.

Patients should never be intrusted during the night to the charge of females alone. The strength of one or two men is sometimes barely sufficient to prevent them from that which would be imminently dangerous to life or limb. They are frequently disposed to jump from windows, from a wharf, &c. with the intention of escaping from imaginary dangers.

During the paroxysm it is well to persuade the patient to lie down several times in the course of the day, as in this way he may secure a little sleep. Toward the end of the third day, this is more important, as the close of the disease is to be then expected, and the position for sleep may in some degree hasten its approach. When the critical sleep has actually taken place, everything which can interrupt it is to be obviated. The patient is to be kept in darkness and silence. Whenever he awakes, as he does occasionally, he may be supplied with drink or nourishment, and encouraged to sleep again.

The treatment after the paroxysm has in it nothing peculiar: the convalescence is generally rapid, and the health better than before the attack.

Very little remains to be said of those cases which fall under the third and fourth classes: it is only necessary to remark, that when we are satisfied that the delirium does not take the place of or suspend a previous disease, we are to proceed in its treatment much as if the delirium had not occurred: when, on the contrary, we believe that it does, we are to manage the paroxysm in the manner which has been now described.

